# Supplementary material for: Altered Cerebral Blood Flow and Potential Neuroprotective Effect of Human Relaxin-2 (Serelaxin) During Hypoxia or Severe Hypovolemia in a Sheep Model
Source: Int J Mol Sci. 2020 Feb 27;21(5):1632. doi: 10.3390/ijms21051632 (PMC7084399; doi:10.3390/ijms21051632)

IJMS-715676 -- Full size blots Figure 9

Assigned molecular weigths (MW) of bands are indicated to the right

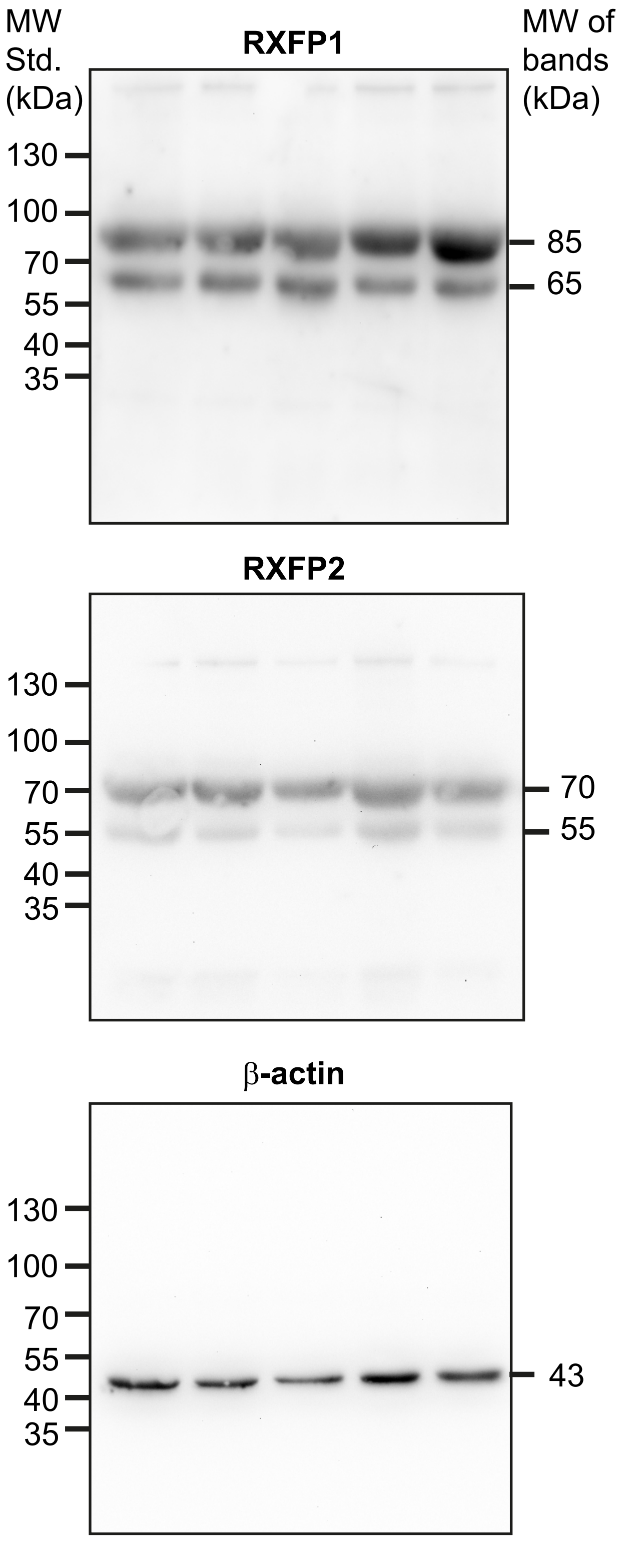

IJMS-715676 -- Full size blots for Figure 10A

Assigned molecular weigths (MW) of bands are indicated to the right

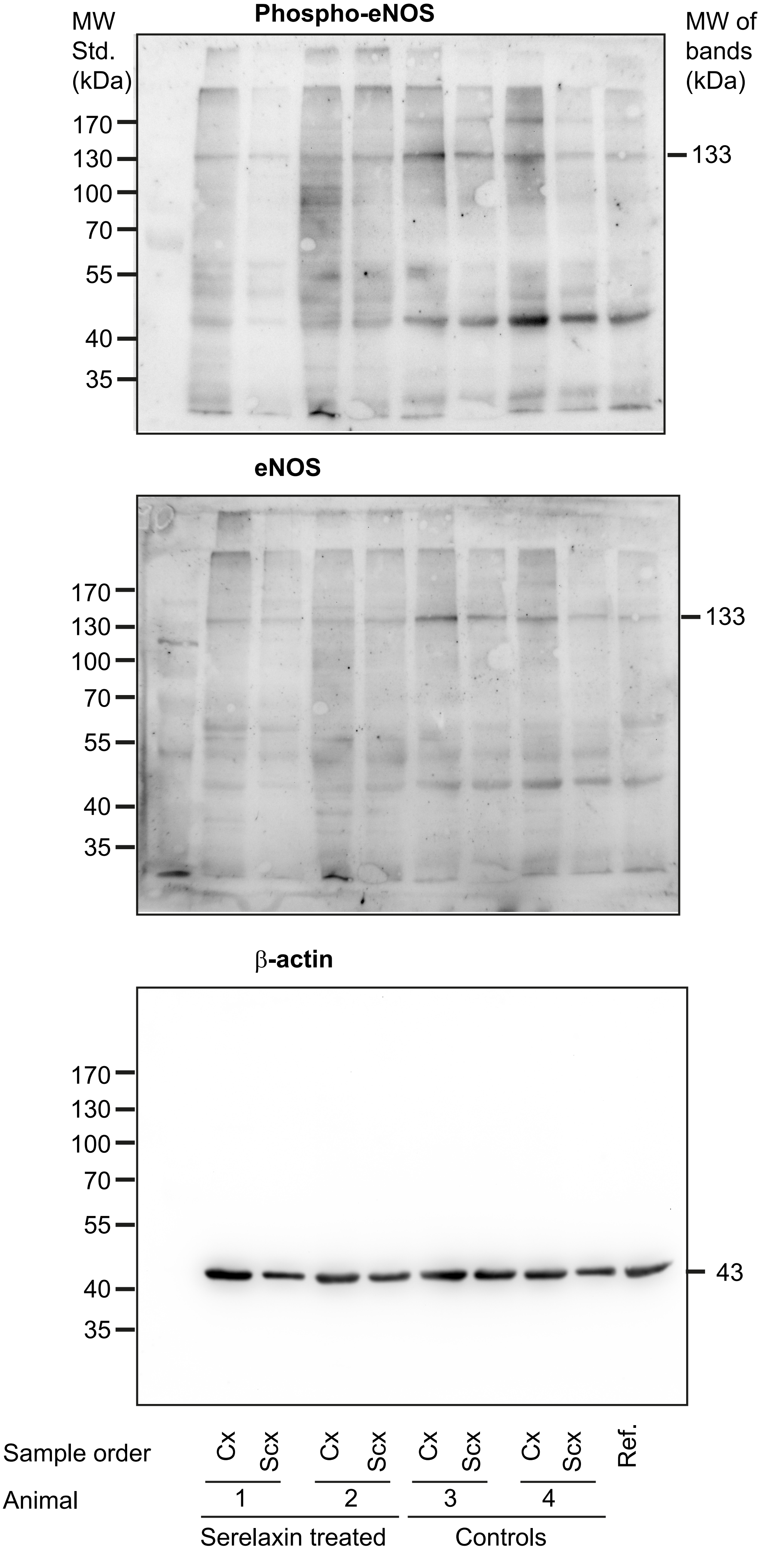

IJMS-715676 -- Full size blots for Figure 10B

Assigned molecular weigths (MW) of bands are indicated to the right

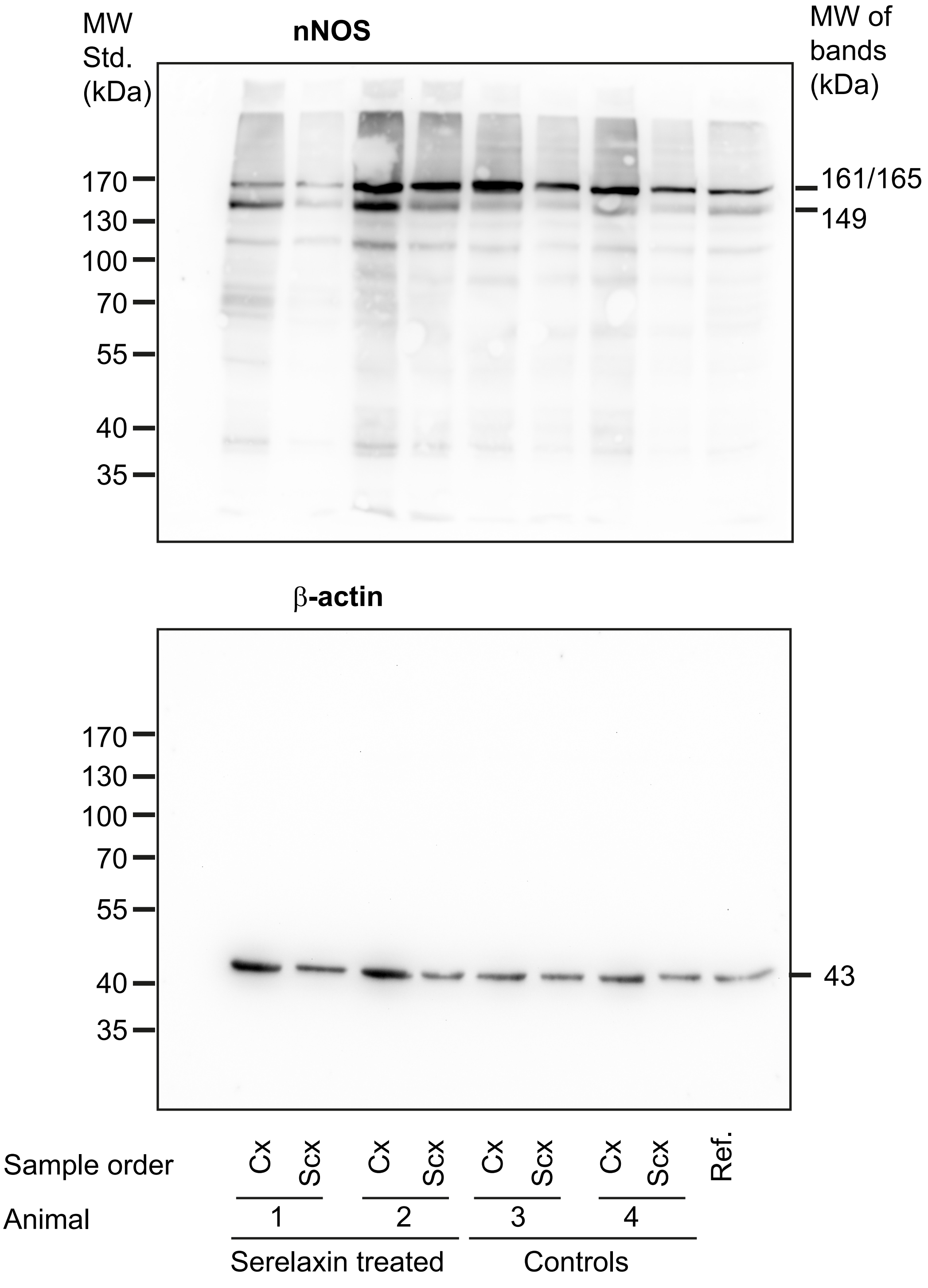

IJMS-715676 -- Full size blots for Figure 10C

Assigned molecular weigths (MW) of bands are indicated to the right

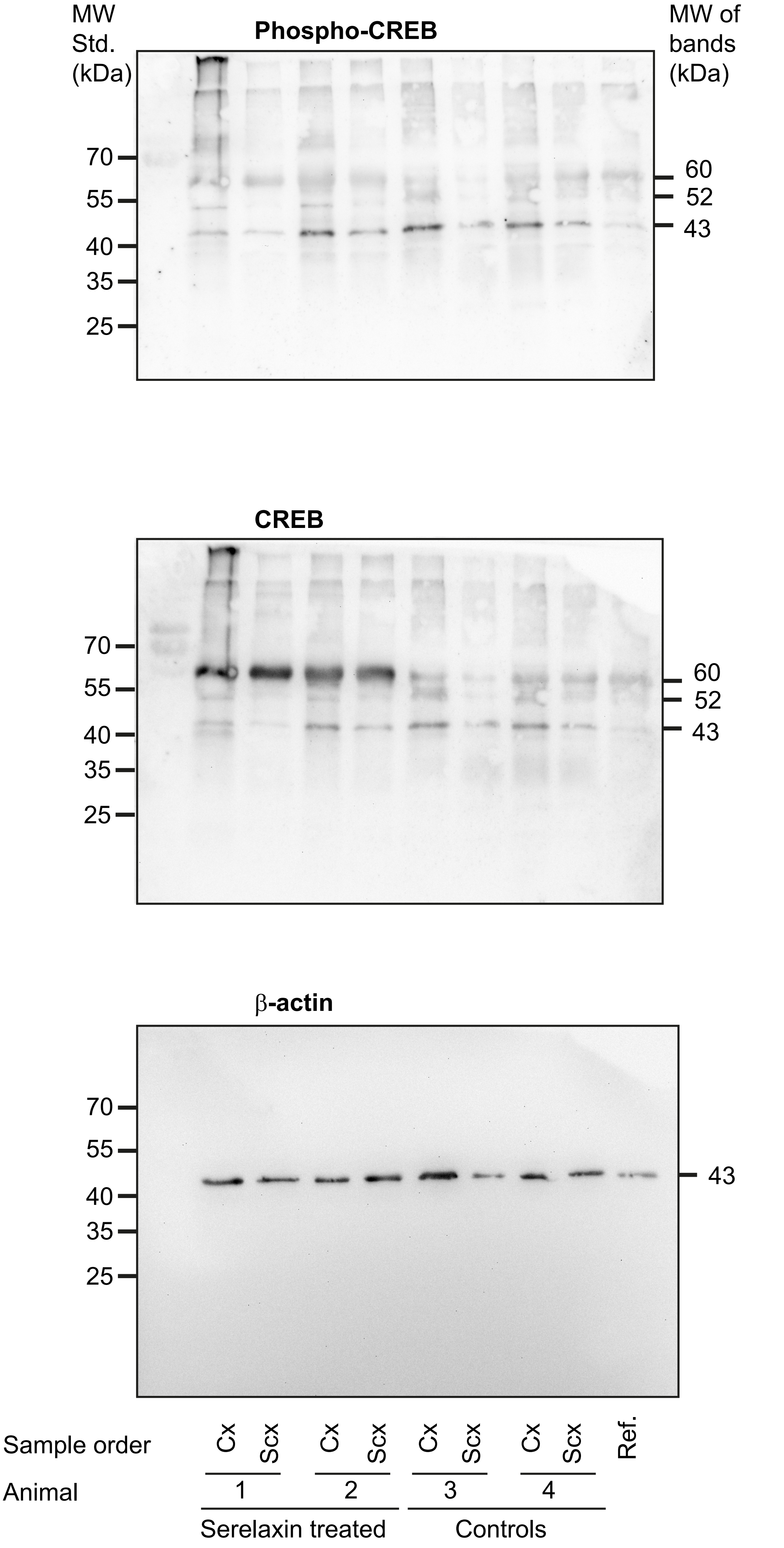

IJMS-715676 -- Full size blots for Figure 10D

Assigned molecular weigths (MW) of bands are indicated to the right

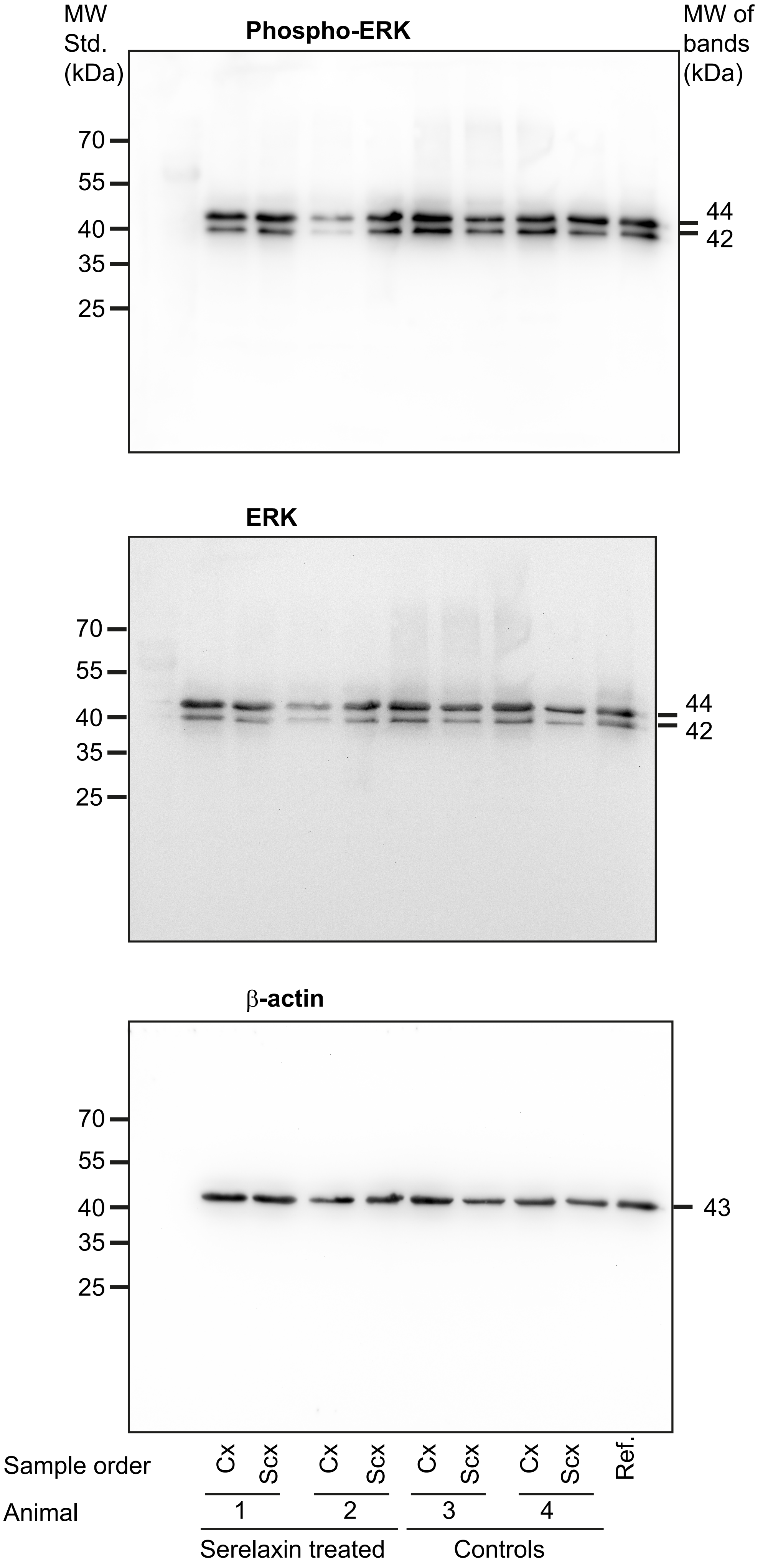

Supplement: Supplementary file 1 [file ijms-21-01632-s001.pdf]
